# The life you never imagined—lived : Life profile of Prof. Dr. Subhash Ranade

**DOI:** 10.1016/j.jaim.2025.101229

**Published:** 2025-10-18

**Authors:** Ankita Abhijeet Shirkande, Abhijeet Sarjerao Shirkande, Gunvant Yeola

**Affiliations:** a Department of Rasa Shastra and Bhaishajya Kalpana (Ayurvedic Iatrochemistry & Pharmaceuticals Science), Dr. D. Y. Patil College of Ayurved & Research Center, Dr. D. Y. Patil Vidyapeeth (Deemed to be University), Pimpri, Pune, India; b Department of Dravyaguna (Ayurvedic Materia Medica & Pharmacology), Dr. D. Y. Patil College of Ayurved and Research Center, Dr. D. Y. Patil Vidyapeeth (Deemed to be University), Pimpri, Pune, India; c International Academy of Ayurved, Pune, India; d Professor, Department of Kayachikitsa, Dr. D. Y. Patil College of Ayurved and Research Center, Dr. D. Y. Patil Vidyapeeth (Deemed to be University), Pimpri, Pune, India

## 1 Introduction

**Fig. 1:**
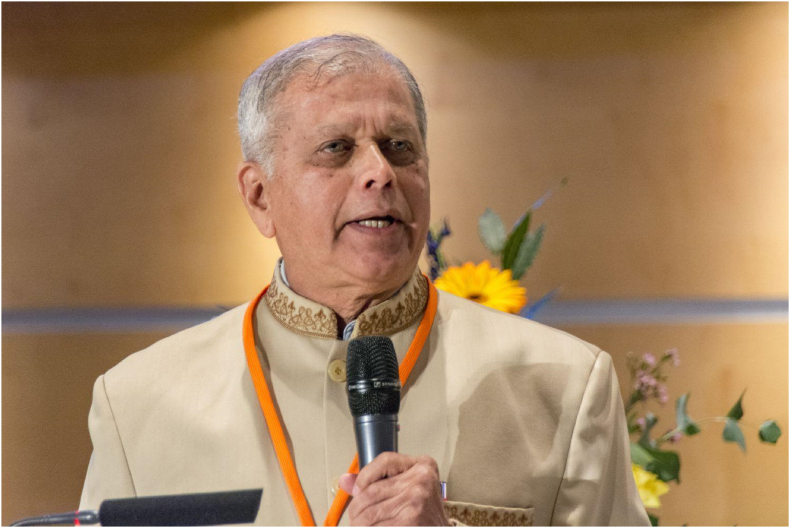
Prof. Dr. Subhash Ranade enlightening the audience at the International Ayurveda Congress (IAVC) London Conference, 2017.

Prof. Dr. Subhash Ranade (1940–2025), renowned as *Dr. International Ranade*, was a pioneering Ayurvedic scholar, global ambassador of Ayurveda and an institution in himself. Over six decades, he contributed extensively to clinical practice, education, research, and international outreach (Fig. 1). His disciplined persona, humility and effective communication left a lasting impact on students and peers alike. Dr. Ranade played a pivotal role in introducing Ayurveda to over 78 countries and authored more than 178 books across multiple languages, covering core Ayurvedic texts, clinical specialties, Panchakarma, women's health, and research methodology. His writings are widely adopted as standard references in Ayurvedic education worldwide. This life profile traces his journey from rural Maharashtra to global recognition, focusing on five themes—education, clinical service, academic leadership, literary contributions, and international propagation, underscoring his legacy in modernizing and globalizing Ayurveda.

## 2 Early life and education

Prof. Ranade was born on June 27, 1940, in Wai, Satara district, Maharashtra, into a scholarly family. His father, Bhalchandra Narayan Ranade, was a municipal school headmaster, and his mother, Nalini Ranade, a teacher in Pune. He completed his B.A.M.S. in 1963 and M.A.Sc. in 1973 from Pune University. In 1960, during his undergraduate studies, he met Dr. Sunanda, whom he married in 1966.

## 3 Ayurveda in action: a fourfold contribution to practice, pedagogy, publication, and progress

### 3.1 Clinical practice and patient care

After graduating in 1964, Prof. Ranade served as Medical Officer at the Primary Health Center in Pawas (Ratnagiri) for six years, treating thousands in rural Maharashtra and promoting preventive healthcare. His clinical practice continued lifelong, including online consultations until his last days. He managed both acute and chronic conditions globally, initially relying on local herbs due to limited resources. He emphasized constitution-based diet, daily and seasonal regimens, and observed significant therapeutic outcomes through these classical Ayurvedic principles.

### 3.2 Academic leadership and teaching legacy

Prof. Subhash Ranade began his academic career in 1968 as Professor at Tilak Ayurveda Mahavidyalaya, Pune, where he served for two decades, mentoring thousands of students. He later became Principal and Professor at Ashtang Ayurveda Mahavidyalaya, Pune, for ten years. In the early years, he taught a wide range of subjects beyond his specialization, strengthening his expertise and supporting his prolific contributions to Ayurvedic literature. He also served as Professor at the Interdisciplinary School of Health Sciences and as Professor and Head of the Department of Ayurveda at Savitribai Phule Pune University. He was appointed as the Visitor's Nominee to Banaras Hindu University by the President of India and designated as the Liaison Head of the International Education Hub by Maharashtra University of Health Sciences, Nashik. He was nominated by the Ministry of AYUSH, Government of India, as a Board Member of the Governing Body of Rashtriya Ayurveda Vidyapeeth, Delhi. Additionally, he held the position of Director at the Ayurveda Rasashala Foundation, Pune, a reputed Ayurvedic pharmaceutical company with a legacy of over 9 years, and serves as Director of the International Maharishi Ayurveda Foundation (Holland). He has also served as Visiting Professor at prestigious institutions globally, including Suddha Dharma Mandalam, Kerala Ayurveda Academy (USA), Internationale Akademie für Ayurveda (Germany), SKA Ayurveda (Italy), Ayurvedic International Diffusing Association (Japan), Ayurveda Prema (Argentina), Aki Sinta Saude (Portugal), and the Israeli Center of Ayurveda, Broshim Campus (Israel), reflecting his significant academic influence in both national and international domains.

### 3.3 A voice of Ayurveda in print and media

Early in his teaching career, Prof. Dr. Subhash Ranade recognized the lack of accessible, high-quality study material for students. Resolving to address this, he set a personal discipline: write at least one page before dinner—or skip dinner. At the time, Ayurvedic education depended largely on untranslated or poorly translated Sanskrit texts, with little subject-wise organization. To bridge this gap, he authored syllabus-oriented textbooks tailored to various academic levels, simplifying complex concepts and aligning content with curricula. His dedication to teaching was reflected in regularly updated editions. By co-authoring and translating key works into multiple foreign languages, he greatly enhanced Ayurveda's global reach and acceptance. His first book on *Padartha Vigyan* was published in 1972, after initial hesitation from publishers. Eventually, Proficient Publications (Anmol Prakashan, Pune) took the first step, followed by support from Chaukhambha Sanskrit Publishers (Delhi), Lotus Press (USA), Suddha Dharma Mandal (Brazil), and others. Since then, he has authored over 178 books on Ayurveda and Yoga, published in multiple Indian and international languages including Marathi, Hindi, Malayalam, English, French, German, Italian, Japanese, Korean, Polish, Portuguese, Spanish, Czech, and Russian (https://ayurved-int.com/books/). He has also published several e-books on Ayurveda via platforms such as www.ayurveda-foryou.com. In addition to his books, he has written hundreds of articles in national and international journals and newspapers, and served as consulting editor for renowned health magazines such as *Light on Ayurveda Journal* (USA) and Journal of Research and Education in Indian Medicine (JREIM**,** India). He contributed to the first Ayurvedic CD-ROM *Dhanvantari* (by SaffronSoul) and participated in the documentary *Ayurveda Unveiled* by Gita and Mukesh Desai (USA). His work has reached broader audiences through numerous television interviews across India and abroad, including in the USA, Poland, Italy, Germany, Brazil, Greece, Romania, and Chile.

### 3.4 Clinical research and industrial consultancy

As early as 1968, when pharmacotherapeutics was not yet a widely explored subject in Ayurveda, Prof. Dr. Subhash Ranade actively engaged in clinical discussions with Dr. Sunanda Ranade, who at the time was serving as a Clinical Research Officer under the Central Government. Their collaborative efforts delved into the deeper understanding of Ayurvedic herbs and their therapeutic applications. At Tilak Ayurveda Mahavidyalaya, Dr. Ranade also led the Clinical Trial Center at Tarachand Hospital, Pune, pioneering clinical research in Ayurveda. His expertise in Ayurvedic formulations extended to the industry sector, where he guided numerous Ayurvedic pharmaceutical companies in product development. Among them was Marico Limited, an Indian multinational company in the beauty and wellness sector, known for iconic brands such as Parachute, Saffola, and Beardo. The company drew on Dr. Ranade's expertise to integrate Ayurvedic knowledge into product development. In 2012, he contributed to a clinical study evaluating a herbal oil featuring Brahmi, Jatamansi, and other herbs for memory enhancement in students. Both Dr. Ranade's were on advisory board of Emami Limited is a leading Indian company focused on personal and healthcare products, with a presence in over 60 countries.

### 3.5 Global contributions to Ayurveda and Yoga

**Fig. 2:**
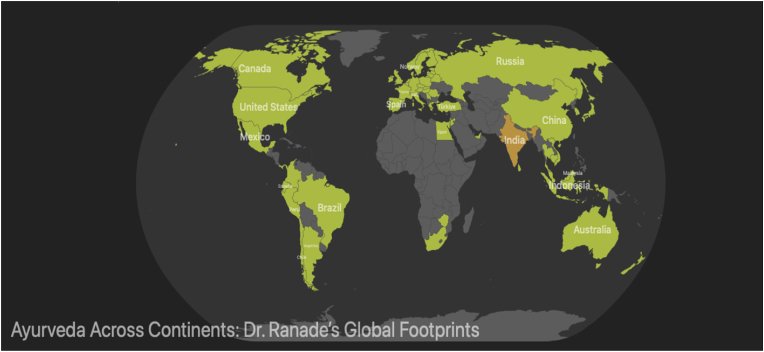
Ayurveda across continents: Dr. Ranade's Global Footprints.

The international dissemination of Ayurveda by Prof. Dr. Subhash Ranade began with a pivotal moment at Ayurveda Rasashala, Pune, when a German manager from the Sterimed Group requested information on Narayan Taila in German. Realizing the significance of language in cross-cultural communication, Drs. Subhash and Sunanda Ranade took the uncommon step of learning German—an unusual academic pursuit in 20th-century India—highlighting their deep commitment to global knowledge exchange. This marked the beginning of their international mission to promote Ayurveda. In 1983, they were invited to Germany for a series of lectures, initiating four decades of global academic engagement. Prof. Ranade subsequently delivered presentations at international forums, including the 1989 conference on traditional medicine in Italy. In 1993, Dr. David Frawley facilitated his seminars across the U.S., training *Heilpraktiker* and influencing students such as Dr. Marc Halpern, founder of the California College of Ayurveda. Over the years, Prof. Ranade conducted structured Ayurveda and Yoga training programs across 78 countries across all five continents, reaching medical professionals, therapists, and the general public. His work significantly impacted regions such as Brazil, USA, Germany, Argentina, Switzerland, Chile, Portugal, Japan, and Canada (Table 1) [1]. Despite considerable logistical challenges like visa constraints, lost baggage, and the manual transport of teaching materials, Prof. Ranade maintained a disciplined travel regimen, often staying abroad for up to six months. His proficiency in multiple languages (German, Italian, Portuguese, and Spanish) enhanced the accessibility of his teachings. His global outreach earned him the moniker “Mr. International Ranade.” Notably, he and Dr. Sunanda Ranade would often coordinate brief personal meetings during international layovers due to overlapping travel schedules. Their extensively stamped passports once led to confusion at immigration desks, where officers presumed they were leading a large group. They held Lufthansa gold cards with air miles equivalent to circumnavigating the globe seven times (Fig. 2). Prof. Ranade's final international engagement occurred in 2022 at the 7th International Ayurveda Congress, Kathmandu, at the age of 82. This phase of his career exemplifies a lifelong commitment to advancing Ayurveda on a global platform through academic rigor, cross-cultural engagement, and exceptional personal dedication.

**Table 1:** Geographic distribution of international Visits [1].

Continent	Region	Country/Constituent State	No. of Visits
**Europe**	Western Europe	Germany (50), Austria (9), Switzerland (6), France (1), Belgium (1), Netherlands (1), Luxembourg (1), Monaco (1), Liechtenstein (1), Ireland (1), England (1), Scotland (1)	74
	Southern Europe	Italy (20), Spain (9), Portugal (4), Greece (1), San Marino (1), Serbia (1)	36
	Eastern Europe	Poland (8), Hungary (3), Czech Republic (1), Slovakia (1), Romania (1), Belarus (1), Lithuania (1), Estonia (1), Latvia (1)	18
	Northern Europe	Denmark (1), Norway (1), Sweden (1), Finland (1)	4
**Asia**	East Asia	China (1), Japan (1), South Korea (2), Hong Kong (1)	5
	Southeast Asia	Singapore (3), Indonesia (2), Malaysia (2), Cambodia (2), Thailand (1), Vietnam (1)	11
	South Asia & Gulf	Sri Lanka (1), UAE (4)	5
	West Asia (Middle East)	Israel (8), Jordan (1), Turkey (1)	10
	Eurasia	Russia (1)	1
**Americas**	North America	USA (25), Canada (2), Mexico (2), Panama (1), Guatemala (1)	31
	South America	Brazil (20), Argentina (5), Chile (2), Peru (1), Colombia (1), Ecuador (1)	30
**Africa**	–	South Africa (1), Zimbabwe (1), Mauritius (1), Egypt (1)	4
**Oceania**	–	Australia (1), New Zealand (1)	2

### 3.6 Establishment of the International Academy of Ayurveda

In response to growing global interest in authentic Ayurvedic education, Prof. Dr. Subhash Ranade and Dr. Sunanda Ranade established the International Academy of Ayurveda (IAA) on January 11, 1996, in Pune, India. The Academy promotes Ayurveda and Yoga through seminars, publications, and structured training programs. More information is available at (https://ayurved-int.com/). Currently, the Academy maintains MOU with nine national and twenty-eight international institutions, attracting students from across the globe for hands-on training in Ayurvedic therapies and procedures. Through strategic collaborations and sustained academic engagement, the International Academy of Ayurveda (IAA) has established itself as a global hub for Ayurvedic excellence and cross-cultural exchange. To date, it has deputed over 100 Ayurvedic practitioners to various countries and welcomed more than 1000 international students to study in India.

### 3.7 A visionary ahead of his time

Prof. Dr. Subhash Ranade exemplified forward-thinking leadership in both personal and professional spheres. At a time when love marriages were socially uncommon, he married Dr. Sunanda Ranade, forming a lifelong partnership rooted in shared values and a commitment to Ayurveda. Together, they co-founded the International Academy of Ayurveda (IAA), among the first Indian institutions to welcome international students for in-depth, classical Ayurvedic study. IAA was also among the first to establish a web presence for Ayurvedic education in India, reflecting Dr. Ranade's prescient embrace of emerging technologies. The Academy emphasized personalized mentorship, assigning students to experienced clinicians and hospitals for practical training-an innovative, decentralized approach that maximized resources long before remote or hybrid education became mainstream. In addition to education, Dr. Ranade placed great emphasis on promoting native ingredients in clinical practice. Dr. Ranade also prioritized the use of locally available herbs in clinical practice, especially during periods when classical Ayurvedic formulations were scarce. He and Dr. Sunanda Ranade often explored herbal gardens, markets, and nurseries, reinforcing the idea that Ayurveda is embedded in everyday environments. Their ethno botanical curiosity even led them to the Amazon rainforest, where they identified numerous herbs described in Ayurvedic texts, making them pioneering Ayurvedic couples to undertake such an exploration. Dr. Ranade particularly valued interactions with tribal communities, recognizing the richness of indigenous knowledge systems and their contributions to the Ayurvedic materia medica. Even in the later stages of his career, Dr. Ranade remained at the forefront of academic progress. When the National Commission for Indian System of Medicine (NCISM) introduced a revised B.A.M.S. curriculum in 2021–22, he promptly revised and published updated textbooks aligned with the new syllabus—continuing his lifelong mission to nurture the next generation of Ayurvedic scholars.

### 3.8 The inner framework of excellence

Prof. Dr. Subhash Ranade's enduring success in Ayurveda was underpinned by personal discipline, spiritual grounding, and a leadership style defined by humility and vision. A natural leader, he fostered collective growth by empowering students and colleagues to take initiative. His punctuality, strategic planning, and clear communication reflected a high level of professionalism. Equally articulate in Marathi, Hindi and English, Dr. Ranade engaged with focused presence, exuding calm and dignity in every interaction. He lived Ayurveda in daily practice—rising early, following Rasayana regimens, meditating, performing Abhyanga and adhering to fixed routines for diet and sleep. Even during international travel, he remained committed to Ayurvedic principles, carrying his own oils for daily rituals. His encouragement of others was heartfelt and sincere, often acknowledging contributions and inspiring confidence. He upheld shared leadership with Dr. Sunanda Ranade, always ensuring her efforts received equal recognition. Spiritually, he remained a disciple of Swami Swarupanand and practiced Transcendental Meditation under Maharshi Mahesh Yogi throughout his life.

### 3.9 A mentor beyond the classroom

Prof. Dr. Subhash Ranade's influence as a teacher and mentor extended far beyond conventional pedagogy. For many students, his unwavering commitment to Ayurveda served as a powerful inspiration to pursue the discipline. His ability to simplify complex subjects like *Padarth Vigyan* and *Ayurveda Samhita Siddhanta* helped bridge the gap for students transitioning from modern science backgrounds. His lucid explanations, relatable examples, and clinical integration made even the most abstract concepts accessible and engaging. Dr. Ranade's mentorship continued beyond the classroom. He was known to attend inaugurations of his students' clinics, hospitals, wellness centers and pharmacies pharmacies offering not just his blessings but genuine encouragement. Even a short visit or phone call from him left a lasting impact, instilling confidence and a sense of belonging. He combined academic excellence with spiritual grounding, fostering not just intellectual growth but holistic development in his students. Many of his students now serve as distinguished clinicians, academicians, researchers, and public representatives. As Director of the Ayurveda Rasashala Foundation, he maintained close ties with leading pharmaceutical houses, collaborating on educational initiatives to promote authentic Ayurvedic practices. Dr. Ranade never missed an opportunity to advocate for Ayurveda in public health discourse. He was equally admired among social workers and community leaders for his efforts to integrate Ayurveda into grassroots health initiatives, especially in underserved regions.

### 3.10 Humanity over commercial success

In 1982–83, when Drs. Subhash and Sunanda Ranade received their first international invitation to lecture on Ayurveda in Germany, financial limitations presented a major obstacle. Though travel expenses were to be reimbursed, they lacked the funds to initiate the journey. In an act of profound commitment, Dr. Sunanda Ranade resigned from her secure government position to access their provident fund, enabling their pioneering international mission. This decision underscored their dedication to the propagation of Ayurveda—not for personal gain, but as a service to global health. Throughout their travels, Dr. Ranade extended support to emerging institutions lacking financial resources but committed to promoting Ayurveda. He often offered his expertise without remuneration or demands, catalyzing the growth of many now-established Ayurvedic centers worldwide. In further service to the field, the Dr. Sunanda and Dr. Subhash Ranade Foundation annually recognizes excellence in Ayurvedic literature by awarding monetary prizes. To date, the Foundation has donated over ₹10 lakh to support scholarly contributions.

### 3.11 Obstacles overcome

Prof. Subhash Ranade encountered several professional and personal challenges throughout his distinguished career in Ayurveda. His pioneering efforts to integrate Ayurvedic principles within modern scientific frameworks were met with resistance from both traditional Ayurvedic scholars and members of the biomedical community. The dissemination of Ayurvedic knowledge to international, particularly Western, audiences was at times subject to criticism, reflecting concerns over cultural adaptation and authenticity. Moreover, institutional support for such global outreach initiatives was limited, necessitating self-directed planning, including the strategic use of academic breaks such as summer and winter holidays to fulfill international commitments. In addition to these professional obstacles, Dr. Ranade navigated personal challenges with notable perseverance. These included acquiring proficiency in the German language to facilitate effective communication during his teaching engagements abroad, balancing a rigorous schedule that encompassed writing, lecturing, clinical consultations, and active mentorship, as well as providing ongoing support for his children's education. While his contributions garnered widespread respect, his work occasionally sparked discourse around the balance between modernizing Ayurvedic practice and preserving its classical roots—a debate that continues to shape the field. These complexities are acknowledged here to present a comprehensive and balanced account of his legacy.

### 3.12 Honors and recognitions

Prof. Subhash Ranade has been the recipient of numerous national and international awards, reflecting his lifelong contribution to Ayurveda. He has received over 8 Lifetime Achievement Awards from esteemed institutions (Table 2).

**Table 2:** Awards received by Prof. Dr. Subhash Ranade.

Sr. No.	Award Name	State/National/International	Presented By/At	Year
1	Lifetime Achievement Award	State	PDE's Ayurveda College and Research Center, Akurdi, Pune	March 2001
2	Lifetime Achievement Award	State	Siddhakala Ayurveda Mahavidyalaya, Sangamner	January 2013
3	Lifetime Achievement Award	State	Madhavbaug Institute of Preventive Cardiology Foundation, Khopoli	March 2014
4	Lifetime Achievement Award	State	Rashtriya Shikshan Mandal, Pune	February 2014
5	TanMan Prerana Puraskar	State	Tanman, Pune	January 2018
6	Sandu Ayurveda Gaurav Puraskar	State	At the hands of Dr. Mohan Bhagwat and AYUSH Minister Shripad Y. Naik, Nagpur	October 21, 2018
7	Vaidya Panchanan Gangadhar Shastri Gune Smruti Puraskar	State	Ahmednagar Co-op. Bank, Ahmednagar	November 25, 2018
8	Lifetime Achievement Award	State	Doctors Directory of India and Z 24 Tass TV, Pune	December 13, 2018
9	Lifetime Achievement Award	State	Maharashtra University of Health Sciences (MUHS), Nashik	June 10, 2019
10	Lifetime Achievement Award	State	Jupiter Ayurveda and Medical College, Nagpur	November 2021
11	Ayurveda Bhushan Award	State	Alumni Association of Government Ayurveda College, Nagpur	November 2021
12	Ayurveda Maharishi Award	State	Ayurved Research and Career Academy, Nagpur	November 2021
13	Nidan Bhushan Award	State	Ayurveda Rognidan Vikriti Vijnyan PG Association, Nagpur	November 2021
14	Best Global Ayurveda teacher Award	State	Lions Club, Nagpur	November 2021
15	Parivartan Ayurveda Jeevan Gaurav Puraskar	State	Presented by Hon'ble Shri Bhagat Singh Koshyari, Governor of Maharashtra	June 2022
16	Gold Medal for Ayurveda work	National	Ayurveda Academy, Vijayawada, Andhra Pradesh	March 2000
17	Pandit Shiv Sharma Oration and Award	National	International Association for the Study of traditional Asian Medicine (IASTAM), Jamnagar	February 2015
18	Fellow of Rashtriya Ayurveda Vidyapeeth (Ratna Puraskar)	National	National University of Ayurveda, Delhi, presented by AYUSH Minister Shri Shripad Y. Naik	May 2017
19	International Dhanvantari Award	International	European Ayurveda Academy, Sydney, Australia	April 2009
20	International Dhanvantari Award	International	AAPNA – Pennsylvania, USA	November 2009
21	Best Ayurvedic Physician Award	International	World Movement for Yoga and Ayurveda, Institute of Sakura take Kan, Spain	May 2009
22	Lifetime Achievement Award	International	Spirit and Nature, Ojai, California, USA	June 2014

### 3.13 Carving a legacy, inspiring generations

Dr. Ranade's journey from rural clinics to global platforms reflects a life shaped by vision and unwavering dedication to Ayurveda. His path led him from modest beginnings to international honors—being featured on global media, hosted as a BCCI guest, welcomed on luxury cruises, and sharing stages with film icons like Dharmendra. At the 9th World Ayurveda Congress, he joined a group photograph with Hon'ble Prime Minister Shri Narendra Modi, symbolizing his national and global recognition [2]. The Government of India commemorated his contributions with a ₹5 postal stamp, a rare honor in the field of Ayurveda. His life serves as a testament that with commitment and belief, even the most modest beginnings can lead to a transformative legacy. His story proves one truth: if he could build such a legacy from scratch, with nothing but dedication and vision—so can you. The world celebrates those who dare to believe in their passion (Table 3) (https://www.youtube.com/watch?v=0soeJUY9VCo).

**Table 3:** Quotes about Prof. Dr. Subhash Ranade.

Sr no	Quote	Quoted By
1	“It has been a true honor to know him and to walk alongside him on this profound journey. He was an exceptional individual—possessing a rare inner peace and deep wisdom, yet radiating boundless energy and dynamism. His presence left an indelible mark, and the impact of his work will undoubtedly resonate for generations to come.”	Dr. Tony Nader, Neurologist, Scientist, President of Maharishi International University, USA
2	"Dr. Subhash Ranade dedicated his life to achieving global recognition for Ayurveda and worked tirelessly toward that noble goal. His scholarly contributions, especially his books, have been widely used—particularly by students in Maharashtra—as foundational study materials. I am confident that his legacy will always be remembered. However, mere remembrance is not enough; it is our collective responsibility to uphold his vision and actively continue the mission of promoting and advancing Ayurveda around the world."	Shri Prataprao Jadhav, Union MoS (IC), Ministry of Ayush & MoS, Health & Family Welfare
3	"For the past 30 years, I have had the privilege of being associated with Dr. Subhash Ranade. He was not only one of the greatest Ayurvedic physicians and teachers in India, but also a respected figure across the globe. His tireless efforts were dedicated to firmly rooting Ayurveda both in its homeland and internationally, with the larger vision of creating a disease-free society."	Padma Shri & Padma Bhushan Vaidya Devendra Triguna, President, All India Ayurvedic Congress
4	"Dr. Subhash Ranade was a true pioneer in spreading Ayurveda to the Western world. As a dedicated teacher, he mentored hundreds of future Ayurvedic educators. He was often the first to offer encouragement—always ready to pat someone on the back for publishing a book or article, and supporting them in every possible way. He cultivated a remarkable global network of Ayurveda friends across all five continents. His life's message was clear: understand your Dharma, your purpose, and pursue it with unwavering dedication and efficiency, just as he did."	Dr. Suhas Kshirsagar, Director, Ayurvedic Healing & Integrative Wellness Clinic, California
5	"I had the privilege of working with Dr. Subhash Ranade for over 40 years. He was the first professor at the Interdisciplinary School of Ayurveda, Savitribai Phule Pune University. We co-authored our first book together and collaborated on numerous television interviews. A lifelong learner, he constantly kept himself updated and deeply engaged with evolving knowledge. His contributions have been foundational—both in advancing the academic field of Ayurveda and in internationalizing its reach and recognition."	Dr. Bhushan Patwardhan, National Research Professor-AYUSH, Former Vice Chairman, UGC
6	"Dr. Subhash Ranade stands as a monumental figure in the field of Ayurveda. Through his exemplary teaching, and tireless global advocacy, he has elevated Ayurvedic education and practice to new heights, inspiring generations and fostering international recognition of this timeless science. Legendary personality in Ayurveda sector."	Dr. Tanuja Nesari, Director, Institute of teaching and Research in Ayurveda (ITRA), Jamnagar

## Author contributions

Conceptualization: AS; Writing-original draft: AS, Writing-review and editing: AbS and GY.

## Declaration of generative AI in scientific writing

No generative AI was used for drafting the manuscript.

## Funding sources

This manuscript did not receive any external funding.

## Declaration of competing interest

The authors declare that they have no known competing financial interests or personal relationships that could have appeared to influence the work reported in this paper.
